# Estimation of linkage disequilibrium and analysis of genetic diversity in Korean chicken lines

**DOI:** 10.1371/journal.pone.0192063

**Published:** 2018-02-09

**Authors:** Dongwon Seo, Doo Ho Lee, Nuri Choi, Pita Sudrajad, Seung-Hwan Lee, Jun-Heon Lee

**Affiliations:** 1 Division of Animal and Dairy Science, Chungnam National University, Daejeon, Korea; 2 Indonesian Agency for Agricultural Research and Development, Ministry of Agriculture, South Jakarta, Indonesia; National Cheng Kung University, TAIWAN

## Abstract

The development of genetic markers for animal breeding is an effective strategy to reduce the time and cost required to improve economically important traits. To implement genomic selection in the multibreed chicken population of Korea, an understanding of the linkage disequilibrium (LD) status of the target population is essential. In this study, we performed population genetic analyses to investigate LD decay, the effective population size, and breed diversity using 600K high-density single nucleotide polymorphism genotypes of 189 native chickens in 14 lines (including Korean native chicken, imported and adapted purebred and commercial chickens). The results indicated that commercial native chickens have less calculated LD (average, *r*^2^ = 0.13–0.26) and purebred native chickens have more calculated LD (average, *r*^2^ = 0.24–0.37) across the entire genome. The effective population sizes of the examined lines showed patterns opposite to those of population LD. The phylogeny and admixture analyses showed that commercial and purebred chickens were well distinguished, except for Rhode Island Red (RIR) purebred lines of NC (NIAS_RIR_C) and ND (NIAS_RIR_D). These lines are difficult to distinguish clearly because they originated from the same respective breeds. The results of this study may provide important information for the development of genetic markers that can be used in breeding to improve the economic traits of native chickens.

## Introduction

The development of phenotype-related genetic markers and causal variants for the improvement of economically important traits facilitates efficient selection in animal breeding. Accordingly, various phenotype-related markers based on microsatellites and single nucleotide polymorphisms (SNPs) have been used in genome-wide association (GWA) and genome-wide linkage studies for the improvement of livestock animals [1–3]. Recently, several high-density marker genotyping platforms have been developed and used in the genomic selection of Holstein cattle and other livestock animals, including chickens [4, 5].

Linkage disequilibrium (LD) is an important population genetic parameter guiding the selection of the best subset of genetic markers associated with causal variants in efforts to achieve high-accuracy marker-assisted selection and to reduce breeding strategy errors in research populations [6, 7]. The extent of LD is an important parameter in GWA studies because it reflects marker distances and inbreeding rates [8]. The extent of LD decreases with increasing marker distance and is influenced by various factors, including migration, genetic drift, mutation, selection, and the recombination rate [9]. Furthermore, this parameter can be used to estimate the effective population size (*N*_*e*_), which describes the demographic status of an observed population [8, 10].

Native breeds currently make small contributions to the market share of animal products, as they are less productive and cost more to feed than commercial breeds. Consequently, the populations of native animal breeds have declined markedly. Recently, however, native animals have attracted attention in recognition of the importance of breed diversity in the face of ongoing climate change and as potential sources of economically important traits [11, 12].

**Fig 1:**
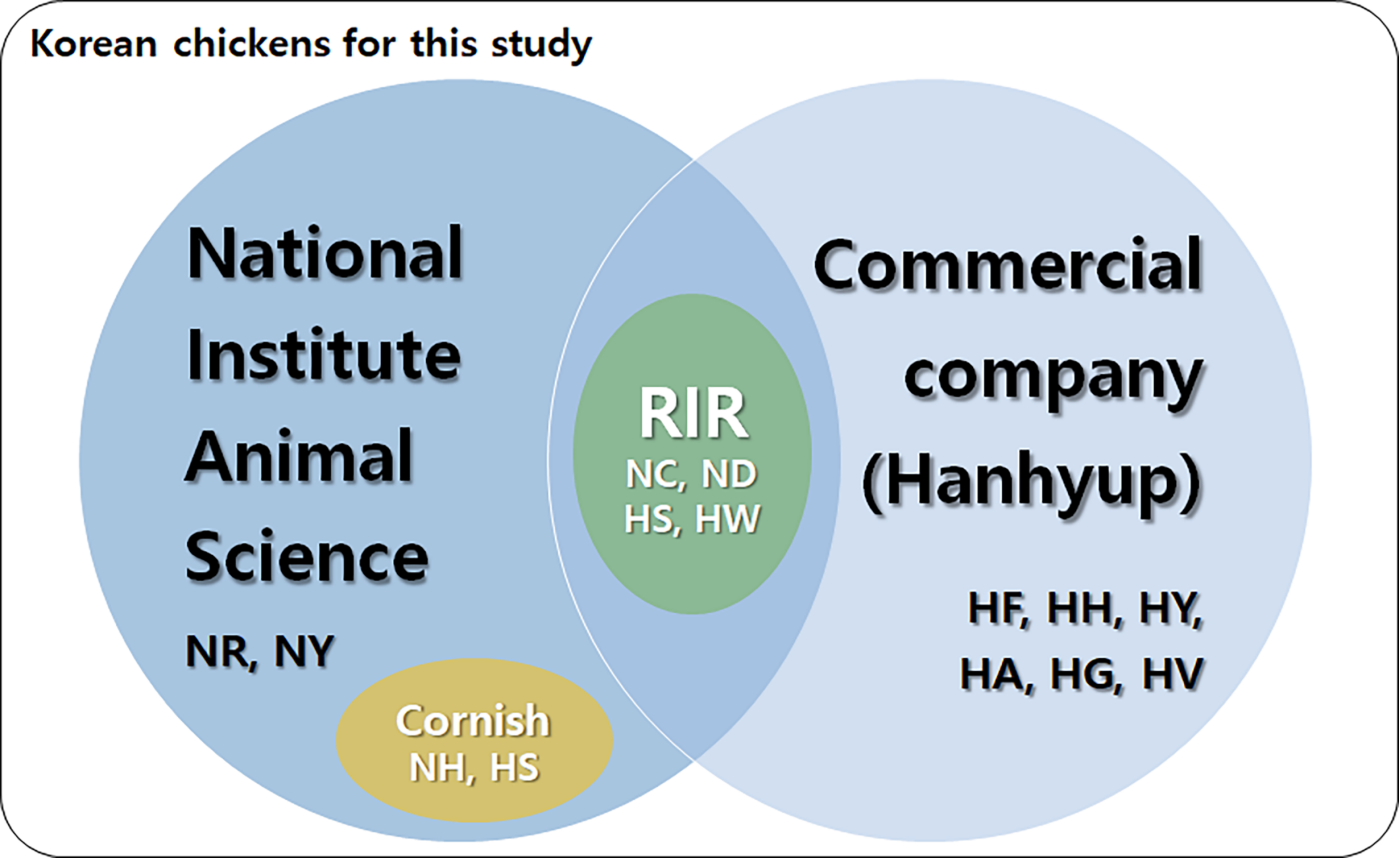
Korean native chicken lines. The National Institute of Animal Science, a government institution, has maintained purebred chicken lines (Korean native chicken: NR, NY) and imported and adapted purebred (IAC) lines [NH, NS (Cornish), NC, ND (Rhode Island Red)]. A commercial company has produced private commercial native chicken purebred lines (HF, HH, HY, HA, HG, HV) and imported purebred lines [HS, HW (Rhode Island Red)].

Korea’s National Institute of Animal Science (NIAS) has preserved two types of purebred chicken line: the purebred Korean native chicken (KNC), which encompasses five lines with different feather colors [red-brown (NR), yellow-brown (NY), gray-brown, black, and white]; and the “imported and adapted chicken” [IAC; two Rhode Island Red (RIR) lines (NC, ND), two Cornish lines (NH, NS), two Leghorn lines (not used in this study)], which was imported in the 1960s for industrialization purposes, adapted to the Korean habitat, and maintained as a purebred line (Fig 1; S1 Table) [13]. The private native chicken breeding-stock company Hanhyup is responsible for more than 80% of native chicken distribution in Korea and has maintained eight of its own purebred chicken lines for commercial use for the past 60 years (S1 Table). After the Korean War, commercial native chicken companies maintained various independent lines while continuing production and market distribution. As these lines have morphological features similar to those of purebred native chicken lines, we expected that the purebred chicken lines might share identical quantitative trait locus-related markers. Identification of these specific markers could aid the selection process in the development of native chickens that are more suitable for the native chicken industry in Korea.

In this context, farmers in Korea have considered commercial production of the KNC, which is known for its adaptability to seasonal changes and the breeding of which is associated with lower costs due to environmental control [14]. The KNC is also recognized to have superior meat quality, with excellent texture and flavor [15, 16]. Korean consumers have been reported to prefer KNC meat to that of commercial broiler chickens due to the former’s amino acid, nucleotide, and collagen contents [17, 18]. Hence, development of the KNC could be commercially advantageous and could provide an important resource for the study of phenotype-related markers in chickens.

In this study, we investigated the genetic diversity, extent of LD, and *N*_*e*_ of KNC populations using high-density SNP data. This genetic information could be useful for the development of genetic markers of economically useful traits of the KNC or for genomic prediction. In addition, it may be useful for the maintenance and utilization of the KNC population and for the establishment of breeding strategies.

## Materials and methods

### Ethical approval

The Institutional Animal Care and Use Committee of Chungnam National University approved this experiment (permit no. CNU-00486). The DNA samples used in the experiment were collected from chicken wing veins based on Assessment of Laboratory Animal Guidelines 1 and 3.

### Sample collection

In total, we used 187 male chicken samples from 14 purebred lines obtained in Korea from Hanhyup and the NIAS (Fig 1; S1 Table.). Samples from eight commercial chicken lines were provided by Hanhyup (HA, *n* = 7; HF, *n* = 23; HG, *n* = 23; HH, *n* = 23; HS, *n* = 23; HV, *n* = 23; HW, *n* = 23; and HY, *n* = 7). The following samples from purebred lines were obtained from the NIAS: NC (*n* = 6), ND (*n* = 6), NH (*n* = 6), NS (*n* = 6), NR (*n* = 7), and NY (*n* = 6). Each group of chickens was nurtured under the same standard feeding and environmental conditions used in a government project performed by the NIAS. Genomic DNA (gDNA) was extracted from blood samples collected from the wing veins into EDTA (ethylene diamine tetra acetic acid)-coated tubes. DNA extraction from the blood was conducted following the standard protocol with a PrimePrep DNA Isolation Kit (GenetBio, Deajeon, Korea). The quality of extracted gDNA was examined by electrophoresis using 1% agarose gels viewed under ultraviolet light. The concentration of gDNA was quantified using a NanoDrop spectrophotometer (Thermo Fisher Scientific, [Waltham, MA], USA). Isolated gDNA samples were stored at –20°C until required for use.

### Genotyping and data filtering

High-density SNP array genotyping was performed using an Axiom 600K chicken array (Affymetrix, [Santa Clara, CA], USA), which contains 580,954 SNP markers identified in the chicken genome [19]. Quality control for genotype data was performed using PLINK software (version 1.9) [20] with the following cleaning parameters: SNPs were removed when the call rate was less than 90%, the Hardy-Weinberg equilibrium *p* value was less than 0.0001, and the minor allele frequency (MAF) was less than 0.01.

### Calculation of linkage disequilibrium

The statistics of linkage disequilibrium (LD) between genome wide SNP pairs was calculated as an *r*^2^ (squared correlation) value among all pairs of syntenic SNP markers. The extent of LD was measured using the following equation:

$$r^{2}=\frac{D^{2}}{freq(A1)*freq(A2)*freq(B1)*freq(B2)}$$

where D = freq(A1_B1)*freq(A2_B2)–freq(A1_B2)*freq(A2_B1), *freq(A1_B2)* is the frequency of the A1_B1 haplotype, *freq(A2_B2)* is a A2_B2 haplotype frequency and *freq(A1_B2)*, *freq(A2_B1)* is a frequency of haplotype A1_B2 and A2_B1 in the population, respectively [21–23]. The *freq(A1)*, *freq(A2)*, *freq(B1)*, and *freq(B2)* were observed frequencies of A1, A2, B1 and B2 alleles, respectively.

The *r*^*2*^ value for all pairs of SNP marker was estimated in Linkage disequilibrium function of PLINK software (version 1.9) [20, 24]. The extent of LD decay across SNP distance was calculated using simple linear regression model (*r*^*2*^ ~ SNP distance + *error*) and was plotted in 28 autosomal chromosome areas with an LD window size of 1,000 kb in R software [25].

To compare the LD structure among breeds, LD correlation was estimated at various marker densities (e.g., 50K, 100K, 150K, 200K, 400K, and 800K intervals), and correlation patterns were compared among breeds.

### Calculation of effective population size

The relationship between *N*_*e*_ and LD (*r*^2^) without mutation was calculated using the following non-linear equation:

$${E(r}^{2})=\frac{1}{4\;N_{e}c+1}$$

Where E(*r*^*2*^) is the expected squared correlation of allele frequencies at a pair of loci, c is the genetic distance between SNP markers in Morgans. Genetic distance (c) was inferred based on the ratio between physical distance of each chromosome and length of the corresponding linkage map. The past effective population (*N*_*e*_) for each population was calculated using the equation [5, 24] as below.

$$N_{e}=\frac{1}{4c}*\left(\frac{1}{r^{2}}-1\right)$$

where *c* is the linkage map distance (in Morgans) and *r*^2^ is the estimated LD value. Then, *N*_*e*_ was fitted by non-linear least squares regression using the R software [25].

Assuming that the population has been constant in size, the approximation of *N*_*e*_ is a value of effective population size for t generation ago, where t = $\frac{1}{2c}$ [5]. That is, *N*_*e*_ is the effective population size at t = 1/2c generation in the past. This equation indicated that LD values at shorter inter marker distances were a value for the estimated *N*_*e*_ in the past generation ago, while markers at longer distances are an estimated value for the recent and current *N*_*e*_ value.

### Genetic diversity analysis

The genetic diversity of the native chicken lines was determined using a multidimensional scaling (MDS) plot, population admixture analysis, and phylogenetic analyses. The MDS analysis was performed in four dimensions using PLINK software (version 1.9) [20], and the higher-effect results (i.e., C1 and C2) were plotted as *x* and *y* coordinates, respectively. Population admixture was analyzed using fastSTRUCTURE software [26], and mean Q values were depicted as a bar plot using R software [25]. Phylogenetic analysis using large amounts of SNP data was performed with SNPhylo software [27]. To investigate relationships among the inferred genetic patterns of populations, we used TreeMix software [28].

## Results and discussion

Experimental chickens from two groups were used in this study. One group consisted of purebred chickens from the NIAS and the other group consisted of chickens from eight private purebred lines from Hanhyup (H lines), which are currently being produced commercially (Fig 1;S1 Table). The LD and genetic variation analyses that were performed in this study provide basic information on the development of genetic markers, which will aid the establishment of breeding and conservation strategies for the Korean chicken population.

### Statistical analyses of genetic markers

In total, we selected 393,418 valid SNPs after quality control of the genotyping results obtained using the Affymetrix 600K Axiom array. The results revealed the occurrence of 69.5% of the total of 580,961 SNPs in the chicken genome. Of these, missing genotype, Hardy-Weinberg equilibrium, and MAF tests led to the removal of 9,314, 132,669, and 35,351 SNPs, respectively (data not shown). Before further calculation, linkage group (*n* = 220) and sex chromosome (*n* = 9,999) SNPs were excluded. Ultimately, 393,418 SNPs on 28 autosomal chromosomes, excluding sex chromosomes and unassigned linkage groups, were used for the genetic diversity and LD analyses. In previous studies, 127,958–473,077 SNPs have been observed in various chicken breeds [19]. Furthermore, the numbers of SNPs on the macrochromosome, intermediate chromosome, and microchromosome were 117,320–187,628, 40,438–63,256, and 55,997–91,505, respectively (Table 1), which is 10 times more than recorded in a previous study of LD in broiler chickens [6]. Therefore, the SNP genotyping results from this study provided a suitable basis for further genetic analyses. The chicken lines examined in the present study differed in coverage of the total of 393,418 SNPs. The HF line had the highest coverage rate (87.03%), and the NC and ND lines had the lowest coverage rates (55.64% and 54.33%, respectively; Table 1).

**Table 1:** Number of valid SNPs on each chromosome type used for calculation of LD in the chicken lines.

	HA	HF	HG	HH	HS	HV	HW	HY	NC	ND	NH	NR	NS	NY
Macro-chromosome	166,545	187,628	163,044	182,504	171,378	152,187	175,633	140,646	121,120	117,320	150,749	168,886	143,438	165,270
Intermediate-chromosome	55,563	63,256	54,102	60,414	55,945	50,085	58,268	44,411	41,421	40,438	52,521	56,244	45,765	55,426
Micro-chromosome	80,964	91,505	78,209	87,487	79,838	72,974	84,233	64,892	56,338	55,997	71,280	81,477	66,992	79,144
Total	303,072	342,389	295,355	330,405	307,161	275,246	318,134	249,949	218,879	213,755	274,550	306,607	256,195	299,840
Coverage (%)	77.04	87.03	75.07	83.98	78.07	69.96	80.86	63.53	55.64	54.33	69.79	77.93	65.12	76.21

NC, ND: Korean Rhode Island Red; NH, NS: Korean Cornish; NR, NY: purebred Korean native chicken; HA, HF, HG, HH, HV, HY: Hanhyup native chicken lines; HS, HW: Hanhyup Rhode Island Red.

### Linkage disequilibrium analysis

**Fig 2:**
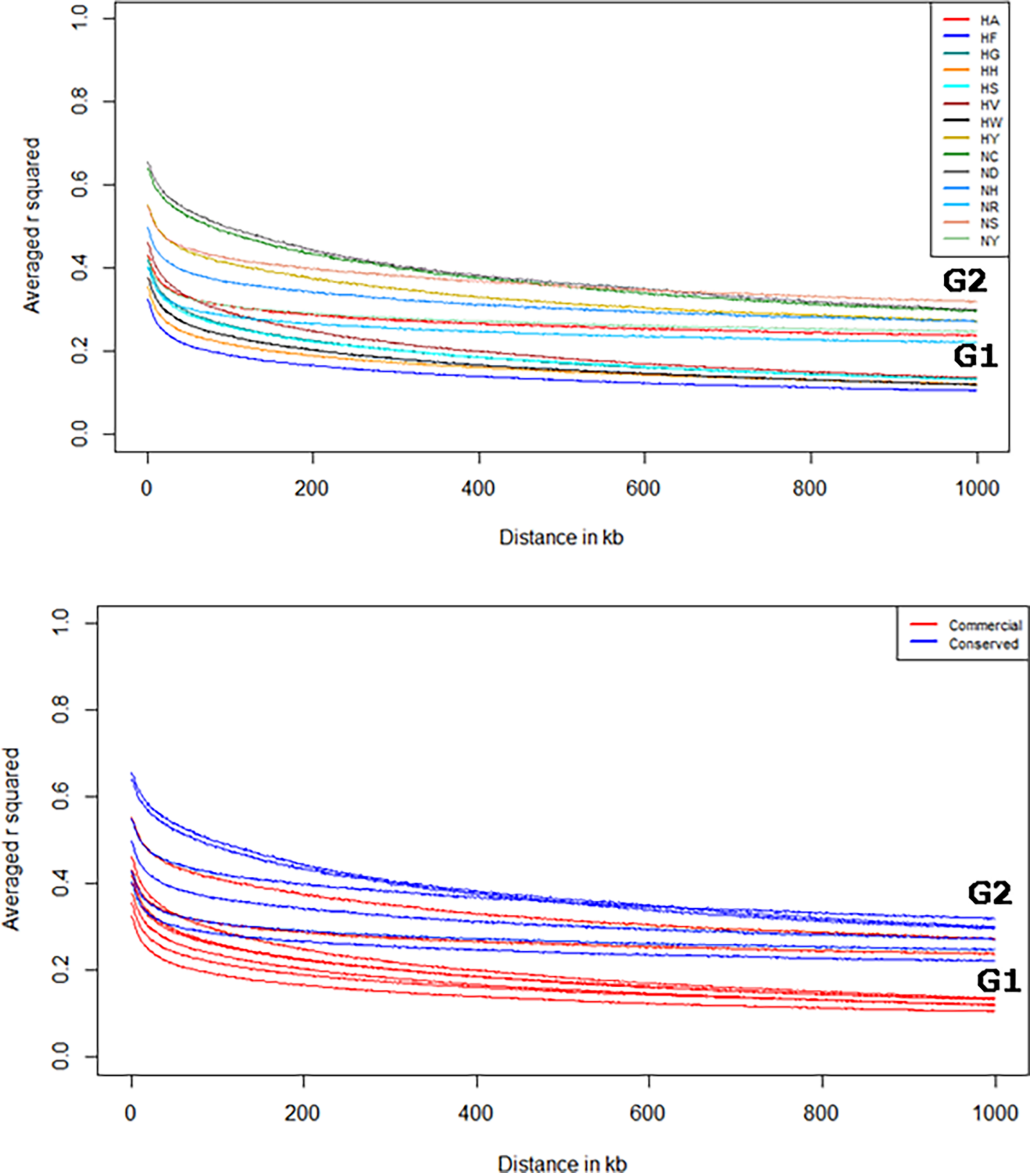
LD decay analysis results (*r*^2^ values of correlation between the closest markers). Inter-marker distances of LD were calculated from 0 to 1 Mbp, and samples were divided into two groups according to converged *r*^*2*^ values: group 1 included HF, HG, HH, HS, HV, and HW, and group 2 included NR, NY, NC, ND, NH, NR, HY, and HA. LD decay was confirmed in each line (a) and compared between the KNC and commercial native purebred lines (b).

To provide comparable LD values for the N-line (NIAS purebred and IAC) and H-line (commercial Hanhyup) populations, we calculated LD values for each population using all SNP pairs and plotted them along 1-Mb distances. Overall, chicken lines with less LD were found to have large numbers of effective SNPs, whereas chicken lines with more LD were found to have smaller numbers of effective SNPs. LD (*r*^2^) values for the whole population were 0.28–0.62 at 20-Kb distances and 0.12–0.34 at 1-Mb distances (Fig 2). HF had the lowest *r*^2^ values (0.12–0.28) over the entire LD distance range. The average LD values for the macrochromosome, intermediate chromosome, and microchromosome were 0.16, 0.14, and 0.12, respectively. The highest *r*^2^ values were obtained for the ND (0.40–0.62) and NS (0.34) lines in the 1-Mb distance range (Table 2). In a previous study, the *r*^2^ values for purebred broiler chickens were approximately 0.34–0.40, and converged at 0.02 in the 5-Mb distance range [6]. A similar trend was observed for the Korean chicken lines, but the extent of LD differed from that observed in the broiler chickens. The *r*^2^ values for native chickens (0.12–0.62) were almost two times higher than those for broiler chickens (0.05–0.12) within the 1-Mb marker interval [6].

**Table 2:** Distribution of *r*^*2*^ values used for estimation of LD according to window size (Kb).

	Fragment						Average
Chicken line	20	50	100	200	500	1000	
**HA**	0.39	0.34	0.32	0.30	0.27	0.25	0.31
**HF**	0.28	0.23	0.20	0.18	0.15	0.12	0.19
**HG**	0.37	0.31	0.28	0.24	0.20	0.15	0.26
**HH**	0.31	0.26	0.23	0.20	0.17	0.14	0.22
**HS**	0.36	0.31	0.27	0.24	0.20	0.15	0.25
**HV**	0.41	0.35	0.31	0.27	0.21	0.16	0.29
**HW**	0.34	0.28	0.25	0.22	0.18	0.14	0.23
**HY**	0.51	0.46	0.42	0.39	0.34	0.29	0.40
**NC**	0.60	0.54	0.50	0.46	0.39	0.32	0.47
**ND**	0.62	0.56	0.52	0.47	0.40	0.33	0.48
**NH**	0.45	0.40	0.38	0.35	0.32	0.29	0.37
**NR**	0.36	0.31	0.29	0.27	0.25	0.23	0.29
**NS**	0.51	0.46	0.43	0.41	0.38	0.34	0.42
**NY**	0.39	0.34	0.32	0.30	0.28	0.26	0.31
**Max**	0.62	0.56	0.52	0.47	0.40	0.34	0.48
**Min**	0.28	0.23	0.20	0.18	0.15	0.12	0.19
**Average**	0.42	0.37	0.34	0.31	0.27	0.23	0.32

NC, ND: Korean Rhode Island Red; NH, NS: Korean Cornish; NR, NY: purebred Korean native chicken; HA, HF, HG, HH, HV, HY: Hanhyup native chicken lines; HS, HW: Hanhyup Rhode Island Red.

Based on the *r*^2^ values, the chickens were divided into two population groups: group 1 (G1) contained five lines with low LD values (HG, HS, HW, HH, and HF), and group 2 (G2) contained nine lines with high LD values (NC, ND, NS, NH, NY, NR, HA, HV, and HY; Fig 2). The two groups showed different degrees of decay in *r*^2^ values due to differences in LD. In G1, the *r*^2^ values converged in the 0.12–0.15 range; in G2, these values converged in the 0.16–0.34 range. The pattern of LD decay detected in the present study is similar to that observed in a previous study of broiler chickens, in which purebred chickens showed greater LD than did crossbred chickens because the latter’s large LD blocks were broken by recombination during cross-breeding [6]. This result implies that the H and N lines examined in the present study had more LD than did the broiler chickens, presumably due to the maintenance of these pure breeds for a long time (Fig 2) [6].

The large extent of LD in the N lines may be explained by two factors. First, differences in sample size might affect the extent of LD. The average sample size for populations in G2 was 23, whereas that for populations in G1 was approximately four times smaller. Indeed, when we re-calculated *r*^2^ values using only six samples from each population, the difference in the convergence of *r*^2^ values between the G1 and G2 populations was smaller (S1 Fig). The converged *r*^2^ value for G1 was higher than in the initial analysis. The second possible explanation involves a higher inbreeding rate in the population of purebred NIAS chickens compared with that among commercial chickens, as the former were restored over a short period of time and have experienced strong selection pressure during the process of pure breed formation. Most N lines that were established early have been maintained with small populations [29]. In the H line, HA and HY have also been maintained in small populations to improve production performance in the near future. We suggest that the extent of LD increased due to the conservation process, which has occurred under conditions of low genetic diversity and with small populations. A similar study of cattle also indicated that the large extent of LD observed in a purebred population was influenced by strong selection to maximize performance related to economic traits [30]. For the same reason, the purebred populations of the N line have greater LD than do the commercial populations of the H line. As mentioned above, the same result was obtained because the HA and HY lines were populations maintained by commercial companies.

**Fig 3:**
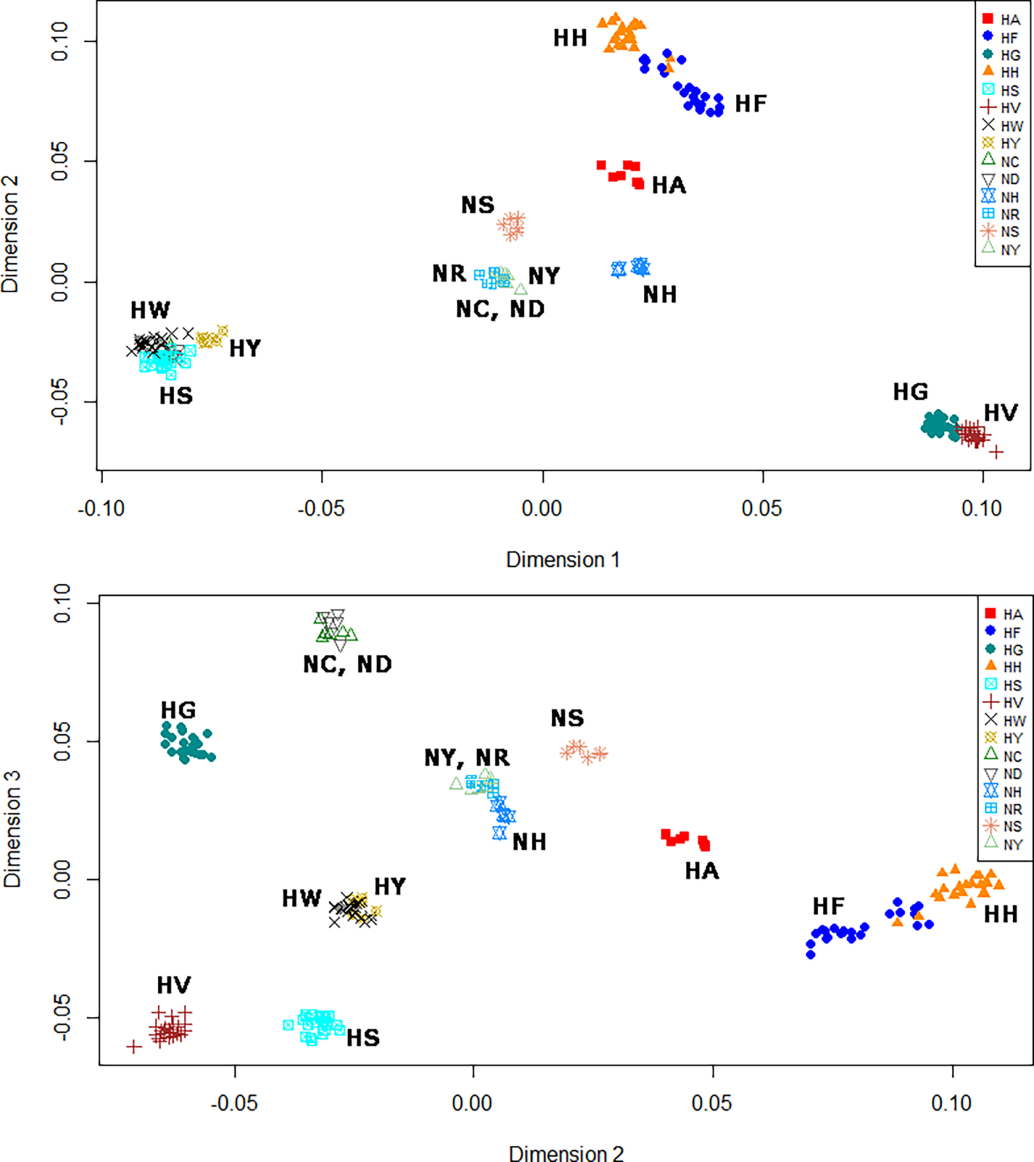
Multidimensional scatter plots of genetic diversity in native chickens, estimated using 600K SNP genotypes. (a)MDS plot result in first and second quadrants, (b)MDS plot result in second and third quadrants.

**Fig 4:**
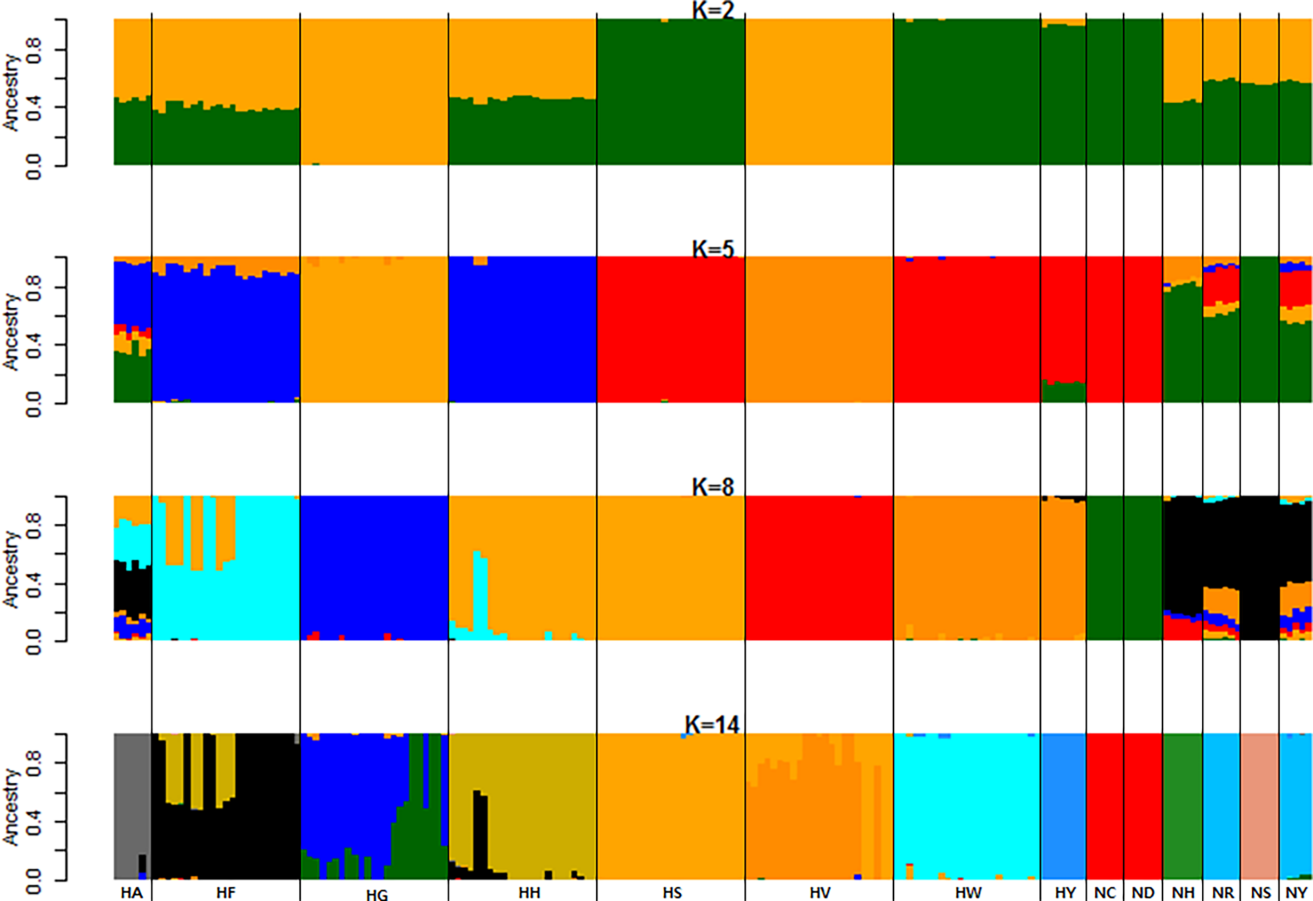
Admixture analysis results indicating haplotype diversity in 14 native chicken lines. The lowest CV error rates were confirmed at *k* = 8. In the bar plot, each bar represents a sample, and the color represents a genetic component.

Interestingly, Hanhyup and the NIAS derived the HS, HW, NC, and ND lines from the same base population of RIR chickens. As mentioned above, these RIR lines were imported and have been maintained as purebred lines by Hanhyup and the NIAS. Because of their greater genetic similarity, the HS, HW, NC, and ND lines shared the same position on phylogenetic branches in the MDS plot, and yielded similar admixture results (Figs 3 and 4). The NC and ND lines had the most LD among the purebred NIAS chicken populations and could not be distinguished clearly; admixture results confirmed that they shared a common ancestor (Fig 4). On the basis of these results, we assume that Hanhyup has maintained large HS and HW populations for production. In previous studies, crossbred populations have shown less LD and more rapid LD decay than have inbred populations for each chromosome type, and these patterns have been confirmed in almost all animal breeds and types [31–33]. In addition, Aerts [34] reported that LD increased with the inbreeding rate and decreased with increasing hybridization. This pattern helps us to interpret the results of the present study.

The complete H and N lines showed a high degree of LD correlation (*r*^2^ = 0.525), and the purebred KNC lines (NR, NY) showed strong correlation with the Hanhyup lines (HA, HH, HF, HY, HG, HV). In addition, when correlations between the purebred KNC line and the Hanhyup RIR, IAC RIR, and IAC Cornish lines were examined, *r*^2^ values of 0.274, 0.194, and 0.221, respectively, were obtained. The weakest correlation was between the IAC RIR and IAC Cornish lines (*r*^2^ = 0.180) (S2 Fig). Interestingly, the Hanhyup RIR and IAC RIR showed a high degree of correlation (*r*^2^ = 0.359), which remained high even as the extent of LD increased (S2 Fig). These results suggest that the two varieties were imported at the same time and have the same origin. The overall LD correlation results show that the N and H lines have similar LD status.

### Estimation of effective population size

**Fig 5:**
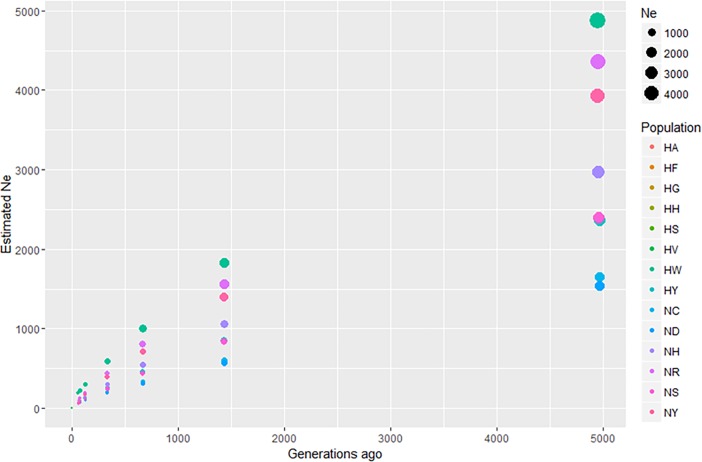
Estimation of effective population size (*N*_*e*_) using LD (*r*^2^) values. The *x* axis shows generations ago (maximum, 5,000) and the *y* axis shows estimated *N*_*e*_.

*N*_*e*_ estimation can aid the identification of changes in the composition of chicken populations caused by genetic drift, the determination of population diversity, and the establishment of selection standards [8, 35]. Approximately 5,000 generations ago, HF had the highest *N*_*e*_ (6,310) and ND had the smallest *N*_*e*_ (1,540). The estimated *N*_*e*_ values for the purebred KNC lines NR and NY were 4,360 and 3,923, respectively (Table 3; Fig 5). Although the IAC N lines and commercial H lines showed similar patterns of decreasing *N*_*e*_ values, the H lines (with the exception of HY and HA) had higher *N*_*e*_ values than did the N lines. The NR and NY lines had higher *N*_*e*_ values than did the other purebred chicken lines, possibly because the purebred KNC lines have been maintained in larger populations than have the other IAC lines at the NIAS, as part of efforts to maintain the KNC lines as representative native chicken populations. The results show a 2.71-fold decline in *N*_*e*_ from 5,000 to 1,500 generations ago and a 1.85-fold decline from 1,500 to 700 generations ago. Furthermore, they revealed a 1.73-fold decrease prior to 300 generations ago and a 2.06-fold decline before 125 generations ago. These changes indicate that the recent decline (that occurring in the past 100 generations) has been more gradual than were those occurring in earlier generations. In addition, the chicken populations of G1 and G2 had different LD decay patterns. The decay was 1.1–1.15 times more rapid in G1 than in G2 before 334 generations ago, and 0.3 times more rapid before 125 generations ago. Overall, the decay observed for G1 was 1.1–1.3 times more rapid than that recorded for G2. This result shows that the *N*_*e*_ of G1 is declining faster than that of G2, and that LD is increasing continuously in G1. Thus, we can assume that the genetic diversity of the varieties is decreasing while G1 is maintained as a small population with no influx of foreign resources. The LD and *N*_e_ patterns of the HY and HA lines also differed from those of the other Hanhyup lines for the above-described reasons. These two lines have different extents of LD than do other commercial chickens in G1, which can be attributed to line maintenance similar to that practiced by the NIAS.

**Table 3:** Estimated effective population sizes (*N*_*e*_), according to LD values.

Fragment	20	50	100	200	600	800	1000
Linkage distance (cM)	0.01	0.03	0.07	0.15	0.4	0.7	0.9
HA	3844	1375	715	394	171	107	87
HF	6310	2406	1320	775	379	265	226
HG	4170	1572	871	522	270	198	172
HH	5483	2069	1125	659	321	223	192
HS	4386	1630	892	529	269	197	172
HV	3498	1323	742	453	246	187	165
HW	4879	1829	1006	596	305	221	192
HY	2364	852	453	258	125	85	71
NC	1647	599	329	198	102	74	63
ND	1540	565	312	189	99	71	62
NH	2968	1054	551	305	136	88	72
NR	4360	1560	806	440	189	118	96
NS	2394	840	435	240	107	69	57
NY	3923	1389	716	390	166	103	83
**Generation**	**4,956**	**1,433**	**668**	**334**	**126**	**71**	**56**

NC, ND: Korean Rhode Island Red; NH, NS: Korean Cornish; NR, NY: purebred Korean native chicken; HA, HF, HG, HH, HV, HY: Hanhyup native chicken lines; HS, HW: Hanhyup Rhode Island Red.

The difference in *N*_*e*_ between G1 and G2 may be attributable to the genetic diversity of populations or the experimental sample size. The *N*_*e*_ values for G2 were estimated using samples that were twice as large as those used for G1; thus, for each generation, the mean value for G2 was higher than the overall mean, and that for G1 was lower than the overall mean. The results could indicate that differences in the extent of LD and genetic diversity between groups were pronounced because the differences between G1 and G2 decreased from 2.4 times to 1.43 times with an increasing number of generations. These assumptions are based on differences in genetic diversity and the extent of populational LD. The G1 populations showed a similar pattern of decline from the ancestral to current populations, although the HF and HV lines showed an approximately two-fold difference in *N*_*e*_ from that estimated for 5,000 generations ago. This result can be attributed to the difference in LD (*r*^2^ values) because the experimental samples for these groups were the same size. The largest *N*_*e*_ value was recorded for the HF line, which had an *r*^2^ value 0.27, and the smallest *N*_*e*_ values were recorded for the HV (*r*^2^ = 0.41) and HG (*r*^2^ = 0.37) lines. For most populations, the *N*_*e*_ value was proportional to LD (*r*^2^ value).

### Genetic diversity of Korean chicken lines, analyzed using SNP information

**Fig 6:**
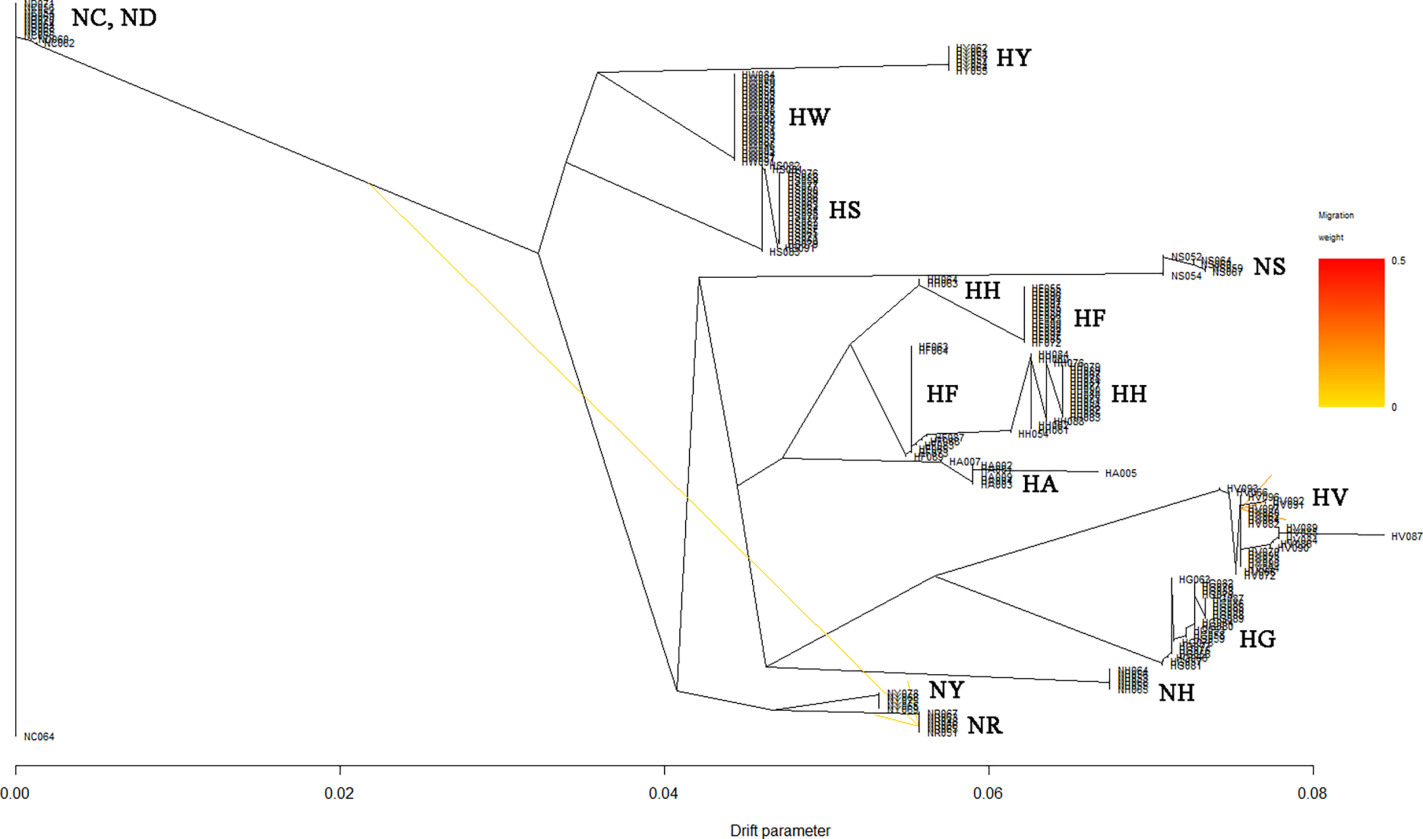
TreeMix results for the 14 native chicken lines. Migration of the chicken population is set to 2, and it can be seen that it starts from the Rhode Island Red of NC, ND population located on the left side and the HV population on the right side, respectively.

We performed MDS, admixture, and phylogenetic analyses using SNPhylo and TreeMix software to examine the genetic diversity of the 14 Korean chicken lines, and differences in diversity among lines. The purebred KNC lines NR and NY were located at the center of the MDS plot, close to NC and ND, in dimensions 1 and 2 (Fig 3A). In contrast, in dimensions 2 and 3, the purebred KNC lines were far away from NC and ND, and close to NH (Fig 3B). The purebred KNC is thus an independent genetic resource, but is related more closely to the NR and NY lines than to other chicken lines. These results suggest that when the purebred KNC was restored in a government project, different lines were distinguished only by feather color. In addition, the purebred KNC line was confirmed to be genetically close to NC, ND, NH, and NS in the MDS and TreeMix analyses (Figs 3 and 6). These results suggest that the purebred KNC line shares an ancestor with the RIR and Cornish breeds, and that it was crossbred with those breeds on farms to improve productivity before restoration. These genetic similarities have also been noted in previous studies of the genetic diversity of KNCs compared with other chicken breeds [35–38]. In particular, studies of the classification of the KNC breed using data from the mtDNA D-loop control region revealed that several KNC haplotypes were also present in the RIR and Cornish lines, indicating the presence of a genetic relationship among these chicken breeds related to origin [39].

**Fig 7:**
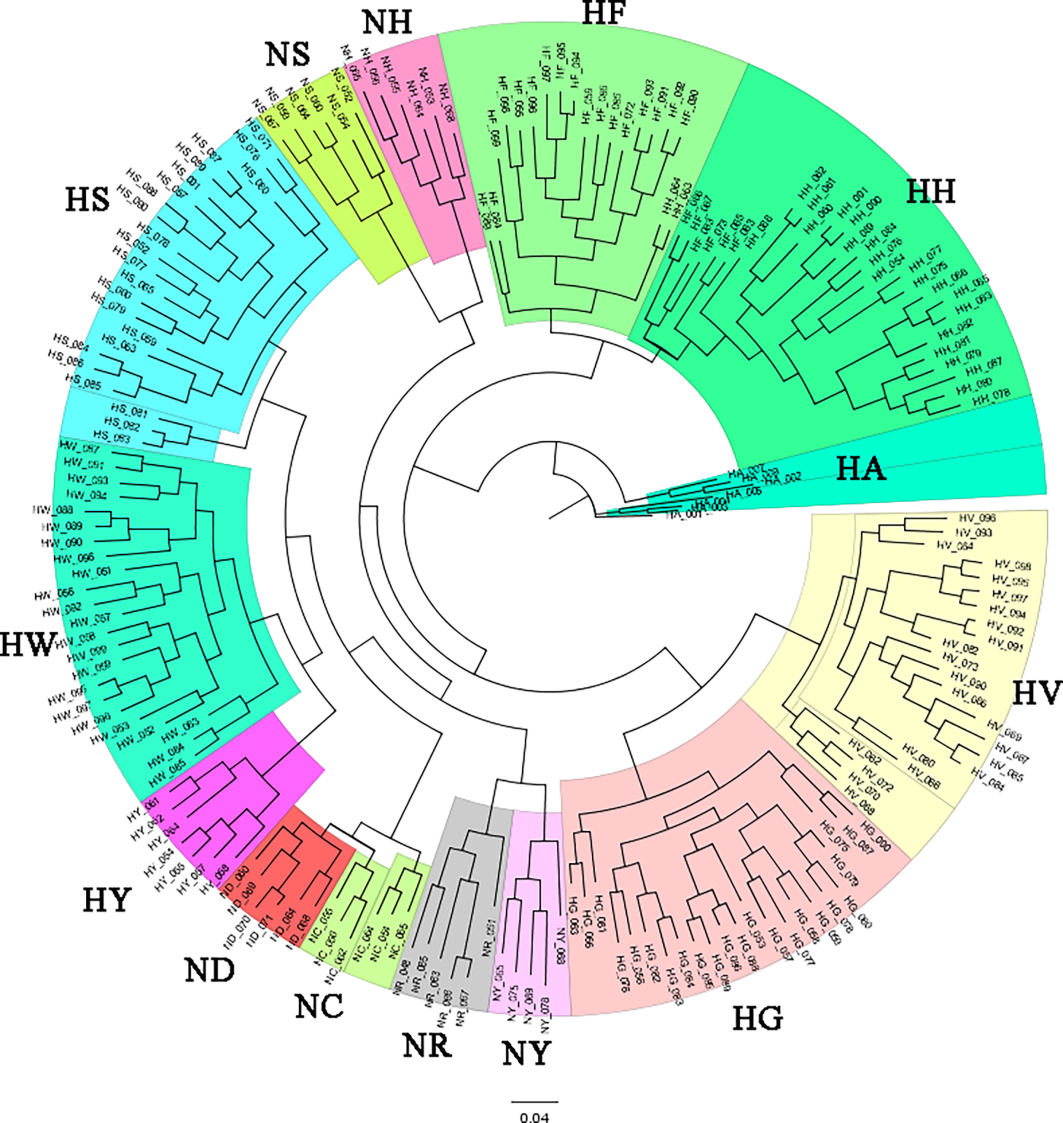
Phylogenetic analysis results for the 14 native chicken lines. Each color block represents one independent chicken line.

NC and ND of the N line, and HS and HW of the H line, are known to be different RIR varieties. MDS and phylogenetic results indicated that NC and ND are clearly distinguishable from HS and HW, but that they share a common root (Figs 3, 6 and 7). However, HS and HW clusters differed distinctly from those of NC and ND. These results suggest that the N-line and H-line RIRs were sorted relatively long ago and have been maintained as different lines, whereas NC and ND were classified more recently as different lines. The findings suggest that HS, HW, NC, and ND share a common ancestor, as reflected even by the *k* = 5 admixture result (Fig 4). In addition, HS and HW were classified into the HS and HW lines at *k* values of 8 and 14, respectively, whereas NC and ND were not divided into two lines; based on these results, one can infer that NC and ND have a greater degree of genetic similarity than do HS and HW (Fig 4). In the SNPhylo and TreeMix analyses, NC, ND, HS, and HW shared the same roots (Figs 6 and 7). Information on the origin of the HY line was not provided, but the SNPhylo and TreeMix results suggest that the HY and HW lines have a common origin. The HY line also showed a high degree of similarity to HW and HS at *k* values of 5 and 8 in the admixture analysis (Fig 4). These results support an RIR origin for the HY line.

In the MDS plot and phylogenetic analyses, HF, HH, HG, and HV were in different areas than were other KNC, RIR, and Cornish chicken breeds. These four lines have been maintained independently. One can infer that the HF and HH lines were classified and maintained relatively recently, as their clusters overlap and are spread widely (Fig 3). In confirmation, two HH samples and six HF samples were identified in each phylogenetic group, and had mutual genetic information in the admixture analysis (Figs 3, 4, 6 and 7). On the other hand, the HG and HV lines were considered to be of the same chicken breed, but divided well into two lines in the phylogenetic and admixture analyses (Figs 4 and 7). Previous studies of genetic diversity in Hanhyup commercial chickens have been conducted mainly with cc-produced individuals, which limits our understanding of common ancestors and ability to determine origin [40]. However, this study revealed genetic diversity, allowing clear distinction of the Hanhyup breeding-stock lines. These results confirm that most Hanhyup chicken lines have been maintained independently, and that the genetic backgrounds of the Hanhyup chicken lines differ from those of the KNC lines.

### Conclusion

In the present study, we analyzed the LD status and genetic diversity of native chicken populations using 600K SNP genotyping. The findings confirm that the purebred NIAS chicken lines have greater LD than do the commercial Hanhyup chickens, and that their *N*_*e*_ patterns are similar to their LD statuses. The two commercial chicken lines maintained for conservation purposes showed greater LD than did the other commercial chicken lines.

As a consequence of genetic diversity, the purebred and commercial chicken lines were found to have distinct genetic differences, and they formed independent clusters in different regions of the MDS plots. Taken together, the results for the NIAS and Hanhyup native chicken resources show that the chicken populations maintained at the two institutions for conservation purposes require continuous evaluation and management of LD and *N*_*e*_ to prevent a decline in genetic diversity. In addition, to enable searches for causal mutations and genes that can be used for the improvement of the chicken lines and development of breeding strategies, the establishment of a reference population and collection of extensive data on genotypes and measurable economically important phenotypes are needed. In addition, the accumulation of useful data on Korean chicken lines and application of genomic selection for rapid improvement are needed. Genetic and phenotypic information accumulated during this evaluation process can be used to search for causal genes and mutations by GWA studies of various economic traits. These results can be expected to provide basic information that can guide efficient improvement of various characteristics of native chickens within a short time.

## Supporting information

S1 Table The basic information of Korean chicken population in this study. (XLSX)

S1 Fig LD decay result (*r*^*2*^ values of correlation between closest markers). Inter-marker distances of LD were calculated from 0 to 1 Mbp, and samples of the same size for each chicken line were divided into two groups by converged *r*^*2*^ values.
(TIF)

S2 Fig LD correlation analysis results for the chicken population. NIAS_Hanhyup: all N and H lines; C_Hanhyup: KNC (NR, NY), Hanhyup (HA, HF, HH, HY, HG, HV); KNC_HRIR: KNC (NR, NY), HRIR (HS, HW); KNC_RIR: KNC (NR, NY), RIR (NC, ND); KNC_Cornish: KNC (NR, NY), Cornish (NH, HS); HRIR_RIR: HRIR (HS, HW), RIR (NC, ND); RIR_Cornish: RIR (NC, ND), Cornish (NH, HS).
(TIF)

S1 File Sup_File1.bed: Genotype information of 600K SNP array in experimental population. (BED)

S2 File Sup_File1.bim: SNP array information of 600K SNP array in experimental population. (BIM)

S3 File Sup_File1.fam: Sample ID information of 600K SNP array in experimental population. (FAM)
