# Supplementary figures and images for: Estimation of linkage disequilibrium and analysis of genetic diversity in Korean chicken lines
Source: PLoS One. 2018 Feb 9;13(2):e0192063. doi: 10.1371/journal.pone.0192063 (PMC5806858; doi:10.1371/journal.pone.0192063)

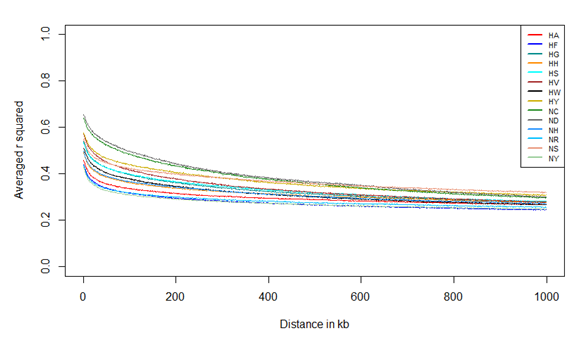

Supplement: S1 Fig — Inter-marker distances of LD were calculated from 0 to 1 Mbp, and samples of the same size for each chicken line were divided into two groups by converged r2 values. (TIF) [file pone.0192063.s002.tif]

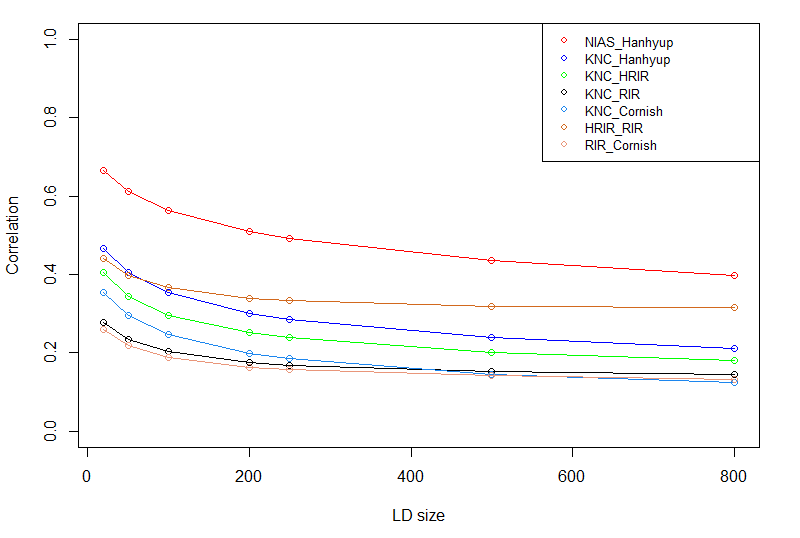

Supplement: S2 Fig — NIAS_Hanhyup: all N and H lines; C_Hanhyup: KNC (NR, NY), Hanhyup (HA, HF, HH, HY, HG, HV); KNC_HRIR: KNC (NR, NY), HRIR (HS, HW); KNC_RIR: KNC (NR, NY), RIR (NC, ND); KNC_Cornish: KNC (NR, NY), Cornish (NH, HS); HRIR_RIR: HRIR (HS, HW), RIR (NC, ND); RIR_Cornish: RIR (NC, ND), Cornish (NH, HS). (TIF) [file pone.0192063.s003.tif]
